# Embedding implementation research to enhance health policy and systems: a multi-country analysis from ten settings in Latin America and the Caribbean

**DOI:** 10.1186/s12961-019-0484-4

**Published:** 2019-10-15

**Authors:** Etienne V. Langlois, Arielle Mancuso, Vanessa Elias, Ludovic Reveiz

**Affiliations:** 1 Alliance for Health Policy and Systems Research, Science Division, World Health Organization (WHO), 20 Avenue Appia, 1211 Geneva, Switzerland; 2 Pan American Health Organization (PAHO), 525 23rd Street NW, Washington, DC United States of America

**Keywords:** Implementation research, co-production, embedding research, health systems research, universal health coverage, policy-making, maternal health, engagement, health systems strengthening

## Abstract

**Background:**

Progress towards universal health coverage requires health policies and systems that are informed by contextualised and actionable research. Many challenges impede the uptake of evidence to enhance health policy implementation and the coverage, quality, efficiency and equity of health systems. To address this need, we developed an innovative model of implementation research embedded in real-world policy and programme cycles and led directly by policy-makers and health systems decision-makers. The approach was tested in ten settings in Latin America and the Caribbean, supported under a common funding and capacity strengthening initiative. The present study aims to analyse ten embedded implementation research projects in order to identify barriers and facilitators to embedding research into policy and practice as well as to assess the programme, policy and systems improvements and the cross-cutting lessons in conducting research embedded in real-world policy and systems decision-making.

**Methods:**

The multi-country analysis is based on the triangulation of data collected via three methods, namely (1) document review, (2) an electronic questionnaire and (3) in-depth interviews with decision-makers. Data from the document review was charted and narratively synthesised. Data from the questionnaire was used to assess three characteristics of the decision-maker’s participation in embedded research, namely (1) level of engagement in different stages of research; (2) extent to which their capacities to conduct and use research were developed; and (3) the level of confidence in undertaking implementation research activities. Interview data was analysed using a thematic approach.

**Results:**

The main barriers to effective delivery or scale-up of health interventions identified in the research projects were inadequate financing, fragmentation of healthcare services and information systems, limited capacity of health system stakeholders, insufficient time, cultural factors, and a lack of information. Decision-makers’ experience in embedded research showed strong engagement in protocol development, moderate engagement in data collection and low engagement in data analysis. The in-depth interviews identified 17 facilitators and 8 barriers to embedding research into policy and systems. The principal facilitating factors were actionability of findings, relevance of research and engagement of decision-makers, whereas the main barriers were time and political processes. In Argentina, the research led to the development of new monitoring indicators to improve the implementation of the perinatal health policy, while in Chile, empirical findings supported the establishment of a training programme on reproductive rights, targeted to municipal health facilities.

**Conclusions:**

This multi-country analysis contributes to the evidence base for the embedded research approach to support health policy and systems decisions-making. Embedding research into policy and practice stimulates the relevance and applicability of research, while promoting decision-makers’ engagement and likelihood to use research evidence in policy-making and health systems strengthening.

## Contributions to the literature

- Improving implementation and scale-up of effective health interventions is critical to support universal health coverage schemes globally.
- Co-production of implementation research by researchers and end-users has the potential to reduce wastage of research and enhance evidence-informed policy and practice.
- Empirical knowledge on the impact and processes of co-production approaches remains limited.
- This multi-country study suggests that implementation research led by decision-makers and embedded into policy and practice stimulates the relevance and applicability of empirical findings, while promoting decision-makers’ engagement and likelihood to use evidence for implementation improvements.
- Key facilitators to embedding research into policy and practice include actionability of findings and relevance of implementation research questions, whereas the main barriers include policy implementation timeframes and complex political processes.
- Embedding research to support implementation raises questions around the competing interests of stakeholders and the need for capacity strengthening, particularly in low- and middle-income countries.

## Introduction

Progress towards universal health coverage and the health-related Sustainable Development Goals requires health policies and systems informed by timely and relevant research. The use of empirical knowledge is a critical component of evidence-informed policy-making [1], yet many challenges impede the uptake of research in health policy development and implementation [2]. Not least is the conduct of research that is non-responsive to policy-makers’ priorities and poorly aligned with the needs of real-world decision-makers [3]. In recent years, various strategies have been developed and tested to bridge the gap between research and policy, largely focusing on knowledge translation, synthesis and exchange. Although some strategies have yielded positive results, conventional knowledge translation approaches have been criticised for failing to adequately consider the complexity of health decision-making processes and the inherent power dynamics [4, 5]. The prevailing practices are necessary but not sufficient to bridge the knowledge-to-action gap and to effectively support evidence-informed decision-making

Greater attention is now provided to the collaboration between researchers and knowledge users, including patients, providers, policy-makers and health system decision-makers [6–8]. There is increasing consensus on the importance of engaging stakeholders, on the premise that co-design and co-production of research has the potential to improve the alignment of science and policy processes and reduce wastage of research [9].

Demand-driven research fosters the relevance of empirical work and increases the propensity of policy-makers to use research findings to support health policy planning and implementation [10, 11]. Yet, most engagement approaches put forth a limited vision of collaboration restricted to dialogues and ad hoc communications [9], and there is a dearth of knowledge on effective stakeholder engagement approaches and determinants [12]. The knowledge gap also concerns good practices to engage policy-makers in the conduct and use of health policy and systems research in various settings, particularly in low- and middle-income countries (LMICs).

In order to improve the policy relevance of research, the Alliance for Health Policy and Systems Research (henceforth ‘Alliance’), an international partnership hosted by WHO, developed an innovative model of research embedded in real-world policy and programme cycles and led directly by decision-makers. The Alliance partnered with PAHO to spearhead the embedding of implementation research in support of health policies and programmes in the Americas. The initiative, entitled Improving Programme Implementation through Embedded Research (iPIER), was implemented in 2014–2015 in Latin America and the Caribbean [13]. One of its crucial components is the focus on implementation research ‘embedded’ (or integrated) in health policy and programme processes. For the purpose of this initiative, ‘embedded’ research corresponds to the engagement of policy-makers and implementers as leaders of the research and their involvement in all phases of the empirical endeavour. The innovative approach placed policy-makers and programme managers in the position of co-principal investigators of the research, with the objective of stimulating demand-driven empirical work [14]. The collaboration is designed to increase the policy-relevance of research questions being addressed and enhance policy-makers’ and implementers’ ownership of research. Policy-makers and health system decision-makers are the actors who are the best positioned to ensure that findings are integrated in real-time to support the development and implementation of health policies and the performance of health systems.

Embedded implementation research aims at scientifically studying the implementation of health interventions, including policies, programmes and services, in different real-world settings and within the existing range of health systems [15]. Engaging closely with decision-makers in a meaningful and timely manner also bears the potential to generate feasible recommendations and increase the use of research to support implementation and health policy-making [6, 9, 16, 17].

The approach was tested in ten settings in Latin America and the Caribbean, supported under a common funding and capacity strengthening initiative. The embedded implementation research teams were selected after an open competitive call for proposals managed by the Alliance and PAHO. The research projects were aimed at supporting existing policies, programmes and interventions using implementation research embedded in real-world decision-making. As such, the focus was not on developing external interventions to enhance the performance of health systems, but rather to use empirical tools and findings to enhance the implementation of active interventions or policies already developed and prioritised by decision-makers.

The teams benefited from training on implementation research and health systems research approaches through a protocol development workshop (Washington, DC, September 2014) and a data analysis workshop (Rosario, Argentina, 2015), where the decision-makers and researchers leading the projects were present. Ongoing scientific and technical support was also provided by the Instituto de Efectividad Clinica y Sanitaria (IECS, Buenos Aires, Argentina), acting as the iPIER technical assistance centre.

The objective of this multi-country study is to analyse embedded implementation research projects in order to identify facilitators and barriers as well as the policy, programme and systems improvements and cross-cutting lessons in conducting research embedded in real-world policy and systems. Our study focuses on ten embedded implementation research projects conducted in eight Latin American and Caribbean countries, namely Argentina, Bolivia, Brazil, Chile, Colombia, Mexico, Peru and Saint Lucia.

## Methods

Over the course of the embedded implementation research initiative, a multi-disciplinary team from the Alliance, PAHO and IECS carried out a continuous monitoring and evaluation of the process of the research endeavours. We periodically collected information on the research activities as well as the use of research findings to improve the implementation of the health policies and programmes under study. The multi-country analysis is based on the triangulation of data collected via three methods — (1) document review, (2) electronic questionnaire and (3) in-depth interviews with decision-makers.

### Document review

We conducted a document review to synthesise and chart the data in relation to the embedded research process and impact of the implementation research. Data was extracted from the research protocol, routine progress reports and the final technical report for each project submitted to the Alliance during their implementation between 2014 and 2016 as well as from scientific papers published in 2017 based on the studies [18–27]. Therefore, five documents were reviewed for each study, making a total of 50 documents, representing all available project documentation from the studies. The document review was conducted by the assessment team (AM, VE) in 2016 and 2017. Data was extracted using a data extraction form that included the headings that are provided in Tables 1 and 2, such as the research question, implementation research variables, implementation research strategies and policy/programme impact, among others. Data extracted were then directly validated in writing on two occasions by the decision-makers and researchers involved in the research in 2016 and 2017. We narratively synthesised the results to highlight key empirical findings and characteristics of the embedded research process as well as policy, programme and health systems improvements.

**Table 1:** Characteristics of embedded implementation research projects in Latin America and the Caribbean

Country	Policy, programme or intervention	Research question	Design and methods	Implementation research variables
Argentina	Policy of regionalization of perinatal health services within the province of Santa Fe	What is the current situation for implementation of the regionalisation strategy in Santa Fe? Which are the main barriers and facilitating factors for policy implementation?	Mixed; Document review, secondary data collection to build process indicators for implementation, and key informant interviews; Delphi method among stakeholder groups; deliberative dialogue, stakeholder analysis	Fidelity, Appropriateness, Acceptability
Argentina	National Chagas Program	What is the best strategy to implement the decentralised distribution of trypanocidal at scale?	Mixed; Secondary data collection, health facility survey, in-depth interviews, focus group discussions	Feasibility, Coverage
Bolivia	Policy of screening for syphilis during antenatal care in Los Andes Health Network	What are the barriers to screening for syphilis during antenatal care?	Mixed; Secondary data collection, in-depth interviews	Coverage
Brazil	Regional program of tuberculosis (TB) control	How to enhance the care of individuals living with TB/HIV co-infection in the setting of specialised care facilities in the state of Ceará, Brazil?	Mixed; Secondary data collection, focus group discussions	Acceptability, Adoption
Chile	Sexual and Reproductive Health Program and National Comprehensive Program for Adolescent Health in the Municipality of Huechuraba	What are the existing problems and shortcomings of the primary care services in Huechuraba that limit adolescents’ access to contraception?	Qualitative; Descriptive study based on participatory action research, which incorporated document review, secondary data collection, and semi-structured interviews with key informants	Appropriateness, Coverage
Chile	National Program for Clinical Practice Guidelines	How can the management of implementation of clinical practice guidelines by the National Program for Clinical Practice Guidelines be optimised?	Qualitative; Semi-structured key informant interviews and focus group discussions	Optimisation, awareness, acceptability
Colombia	Clinical practice guidelines for sexually transmitted infections in Antioquia and Cundinamarca States, Colombia	How does acceptability, perceived usefulness and uptake of implementation tools impact the implementation process of clinical practice guidelines in the Colombian health system?	Mixed; System mapping, surveys, semi-structured interviews and stakeholder analysis	Adoption, Uptake, Acceptability, Perceived usefulness
Mexico	TeleHealth Program in public health services in Oaxaca	What is the process and logistics for implementing TeleHealth (through teleconsultations) in Oaxaca? What is the fidelity of the programme and how can it be improved? What are the requirements to institutionalise the programme?	Mixed; Descriptive study, document review of manuals and management reports, secondary data collection, survey and interviews	Fidelity, Adoption
Peru	National strategies for HIV/AIDS and tuberculosis	What are the barriers to integration of services for HIV/AIDS and TB?	Mixed; Key informant interviews and secondary data collection from TB and HIV registries of the healthcare facilities	Appropriateness, Coverage
Saint Lucia	Modernized newborn screening program for sickle cell disease	What are the barriers to administering neonatal heel prick screening for sickle cell disease sickle?	Mixed; Survey and focus group discussions	Acceptability, Coverage

**Table 2:** Embedded implementation research findings and health policy/programme impact

Country	Results	Implementation strategies	Dissemination methods	Policy/programme impact
Argentina	Implementation of the of perinatal health regionalisation strategy is heterogeneous across different sub-regions of the province; communication among all stakeholders and organisation of transport between levels were identified as the main barriers; general agreement with the strategy is a potential facilitator	Action plan to enhance monitoring and improve implementation	Policy brief; deliberative dialogue	Involvement and participation of key stakeholders from the five sub-regions; establishment of new process indicators to monitor implementation of the regionalisation strategy
Argentina	Major obstacles in the implementation of the National Chagas Program included little articulation between stakeholders, lack of training, difficulties in follow-up of patients and barriers associated with access to services	Decentralisation strategy	Report; website communications	A pilot of decentralisation was carried out, evaluating strategies to optimise intervention on a large scale; results will be reintegrated in scale-up activities
Bolivia	Although healthcare workers believe 100% of pregnant women should be screened, only 55% of the reviewed clinical records indicate syphilis laboratory results and only 37% of perinatal medical histories are reporting the syphilis laboratory results; barriers to syphilis screening included insufficient time for staff to raise awareness among pregnant women about the benefits of screening for syphilis and other diseases, and gaps in communication between medical and laboratory staff	Action plan for periodic review of the coverage of syphilis testing in pregnant women	Workshops and meetings with key stakeholders, including health services providers of Los Andes Health Network, and coordinators of health networks of El Alto rural and urban areas of La Paz Department; discussions on syphilis testing coverage in pregnant women	Strengthening the clinical records and registry; follow-up and monitoring
Brazil	Barriers of tuberculosis (TB)/HIV co-infection management included lack of knowledge of clinical protocols, insufficient human resources, low commitment to address the two diseases, differences in recommendation, e.g. frequency of visits for TB/HIV, and oversubscription of specialist services	Monitoring strategy for integrated TB/HIV care	Results were discussed with managers and health promoters of the HIV/AIDS/hepatitis and TB programmes	Development of a protocol of TB medication adherence
Chile	Adolescents reported difficulties accessing the centres’ contraception services due to cultural factors, lack of information, administrative requirements and existing bureaucratic practices; there were errors in professionals’ management of standards and legal procedures concerning fertility, and no existing interpretive framework recognising sexual and reproductive rights to guide actions; adolescents’ needs associated with their rights are invisible and unsatisfied, and health professionals do not share common criteria for addressing this topic with adolescents	Strategies to improve the programme, in terms of professionals’ training, process of designing and implementing a protocol outlining how to access contraception needs, and active incorporation of adolescents’ feedback	Presentation of results in the Study Local Committee and incorporation of new ideas arising from the Study Coordinator’s participation in the intersectoral network of the Municipality of Huechuraba Departments of Youth and Education, which was formed to implement public policies focused on adolescents	The findings prompted the creation of a training programme on gender and sexual and reproductive rights for professional teams from health centres in the municipality; additionally, a protocol outlining adolescents’ access to health services will be designed and established; the results of the study also contributed to the planning process of a safe space for adolescents, outside of the clinical setting, and the formation of an intersectoral network focused on public policies concerning adolescents; these actions served to address the identified obstacles and improve the quality of programme offerings directed toward adolescents’ sexual and reproductive rights
Chile	The main challenge was lack of a structured process for development and management of clinical practice guidelines; the major strategy recommended to overcome this challenge was the development of an optimised workflow for the development and implementation of clinical practice guidelines, tailored to the Chilean context	Workflow, including strategies, flowchart, management protocols, handbook for clinical practice guideline development	Workshops, publications, staff meetings	Eighty clinical practice guidelines were updated by of the Ministry of Health using the GRADE methodology; administrative changes included transferring the current department to the Division of Health Planning and merging with the Health Technology Assessment Department; an internal document was created to guide the roles and functions of the clinical practice guidelines coordinator within the Ministry of Health
Colombia	Of those who responded to the survey, 86% knew about the clinical practice guidelines (CPG), 86% prioritised the CPG recommendations, 82% used the factsheets and 79% used the interactive flowcharts; 41% had never used the implementation tools; of those who had used the implementation tools, 55% used them on desktops at their work, 24% on smartphones and 21% elsewhere; the most useful implementation tool was the factsheet (98%), followed by interactive flowchart (98%) and prioritised recommendation (92%)	New or revised tools to support implementation	Tools will be disseminated through the Colombian Health Technology Assessment Institute (IETS) web page, conferences and direct technical support to hospitals and other health services providers	Implementation tools will be fine-tuned based on the feedback received; preferred tools will be prioritised
Mexico	The internet connection bandwidth hampers information exchange for teleconsultations; the specialties most in demand are internal medicine and gynaecology; areas for improvement were identified in the programme’s process manuals	Online courses; manual for implementation and operation of TeleHealth program; refresher training; contingency plan for information technology failure; induction programme	Meetings; workshops; presentations	The activities of the Telehealth Coordination (THC) were integrated in the strengthening strategy of the networks of medical units in the state. The THC in Oaxaca was recognised in the Internal Rules of the Health Services of Oaxaca. It increased the number of rural medical units incorporated into the Telehealth Care Network. The schedule of medical specialists of the internal medicine service was extended and is now available on Saturdays, Sundays and holidays
Peru	Barriers identified: little or no coordination between TB and HIV teams, management of the TB/HIV co-infected patients at different levels of care, inadequate financing, scarce or poorly trained human resources and the absence of an integrated information system	Strategy for HIV/AIDS and TB co-infection; technical document to regulate TB/HIV joint activities	Meetings, training sessions	Development of a TB/HIV integrated care model; changes to regulatory framework; planning of joint activities
Saint Lucia	High acceptability of the heel prick (HP) test; the majority of healthcare workers were familiar with the HP test (85.7%) but 74.3% and 72.9% had not attended training sessions on the procedure or the collection of the sample, respectively; a total of 92.9% reported that the HP test was useful; regarding safety, 81.4% felt that the test was not harmful to babies; healthcare workers reported that the test was painful for the baby (74.3%) and 58.6% felt uncomfortable doing the test	Strategy for implementing newborn blood spot screening programme	Presentations and reports; meeting with steering committee	Results have not yet been reintegrated into the screening programme

### Self-administered questionnaire

A standardised electronic questionnaire that contains a series of Likert scale questions was sent to ten decision-makers that were purposefully selected for their involvement in the embedded implementation research projects. Respondents were approached to participate in the questionnaire through email including a link to the survey. Eight respondents completed the survey during January 2016, leading to a response rate of 80%. The survey included 28 Likert-scale questions to assess (1) the level of engagement in different stages of the embedded research; (2) the extent to which their capacities to conduct and use research was strengthened; and (3) the level of confidence in undertaking implementation research activities. All survey responses were included in the analysis.

### In-depth interviews with stakeholders

We conducted post-hoc in-depth interviews with decision-makers who were purposefully selected for their involvement in the research projects. Respondents were approached through email and invited to participate in the interviews. A semi-structured interview guide was used that included a series of open-ended questions on the experience and perceptions of carrying out the embedded research projects throughout the research process, including facilitators and barriers. The questions for discussion during the interviews are presented in Additional file 1. The thematic areas under consideration included (1) conceptualisation and conduct of the research, (2) embedded research approach, (3) uptake of research findings and resulting impact, and (4) perceptions of research.

One interview with each decision-maker (*n* = 10) was conducted by at least two members of the assessment team (AM, VE, LR) using teleconference services, at a time and place agreed upon with the respondent — in most cases, their place of work during working hours. In some cases, the researcher participated in the interviews. Prior to starting the interview, AM had no previous relationship with the respondents, while VE and LR were involved with management of the initiative at the time of the study. The assessment team has received training in qualitative approaches and methods through their education and employment. Respondents were provided with information about the research, including the purpose and reasons for doing the research, prior to obtaining oral consent to participate. Each interview lasted approximately 1 hour and took place between November 2015 and April 2016. The interviews were audio recorded, directly transcribed and translated into English. Notes were taken and recorded during the interviews to summarise and ensure understanding, emphasise important points made during the interviews and identify areas for follow-up questions. The translated transcriptions were uploaded into Nvivo 11 for data management and analysis. Interview data was analysed and coded by one member of the assessment team (AM) using an inductive thematic approach to identify emerging themes derived from the data on the facilitators and barriers to implementation of the embedded research projects [28, 29]. We also put forth a participant validation approach by sharing the results and discussion sections of the study with the country respondents in 2018 to explore the credibility of findings and check for resonance with their experiences. Given the small sample size, data saturation was not assessed as all decision-makers engaged in the initiative were approached and the findings are meant to contribute a spectrum of experiences and perceptions for further discussion as the embedded research approach is becoming more common. Qualitative findings were reported following the Consolidated criteria for reporting qualitative research (COREQ) guidelines (see Additional file 2). This study was approved by the Ethical Review Committee of PAHO and WHO (PAHO-2016-02-0005).

## Results

### Document review

The characteristics of the embedded implementation research projects are provided in Table 1, which presents the validated results from the document review, and a summary is provided below. The majority of research projects were conducted at national level and consisted of mixed methods implementation research studies. The key issues studied focused on maternal and child health and infectious diseases, for instance, the integration of health services to prevent and control co-infection by tuberculosis and HIV. Table 2 summarises the implementation research findings, implementation strategies, dissemination methods and impact at policy, programme and systems-levels.

The majority of studies identified strong impediments to implementation, with the main barriers to effective delivery or scale-up of health interventions identified being inadequate financing, fragmentation of healthcare services and information systems, limited capacity of health professionals and health system stakeholders, insufficient time, cultural factors and lack of information. The principle facilitators reported were adequate communication incentives, training of human resources, and existence of an appropriate implementation strategy. All studies produced improvement strategies supported by the research findings, including implementation strategies (*n* = 5), operationalisation improvements (*n* = 4) and action plans (*n* = 2). Health policy and programme improvements involved these evidence-informed products to optimise the implementation and scale-up of health interventions.

The majority of studies reported positive impacts on the implementation of health policies and programmes in their respective health systems settings. In Argentina, for instance, implementation research conducted on the perinatal health regionalisation policy, along with a deliberative dialogue discussing the research findings, led to the development of new monitoring indicators to support policy implementation [20]. In Chile, the findings of implementation research to improve access to contraceptive services among adolescents informed the development of a training programme on gender, sexual and reproductive rights for professionals working in municipal health facilities [25].

### Questionnaire findings

The questionnaire was circulated to all decision-makers involved in embedded research projects and 8 out of 10 decision-makers responded. Table 3 shows the characteristics of questionnaire respondents. Briefly, the respondents included 4 men and 4 women, and were from Argentina, Chile, Colombia, Mexico, Peru or Saint Lucia. The respondents were from the national (*n* = 4), provincial/state (*n* = 3) and municipal (*n* = 1) levels.

**Table 3:** Questionnaire respondent characteristics

Country	Affiliation	Level	Sex
Argentina	Direction of Child, Adolescence, Sexual and Reproductive Health, Ministry of Health of the Province of Santa Fe, Argentina.	Provincial	Male
Argentina	National Chagas Program	National	Female
Chile	Municipality of Huechuraba	Municipal	Female
Chile	Ministry of Health	National	Female
Colombia	The Colombian Health Technology Assessment Institute (IETS)	State	Male
Mexico	Centro Nacional de Excelencia Tecnologica en Salud (CENETEC-Salud) Servicios Estatales de Salud de Oaxaca	State	Male
Peru	Ministry of Health	National	Male
Saint Lucia	Ministry of Health	National	Female

**Fig. 1:**
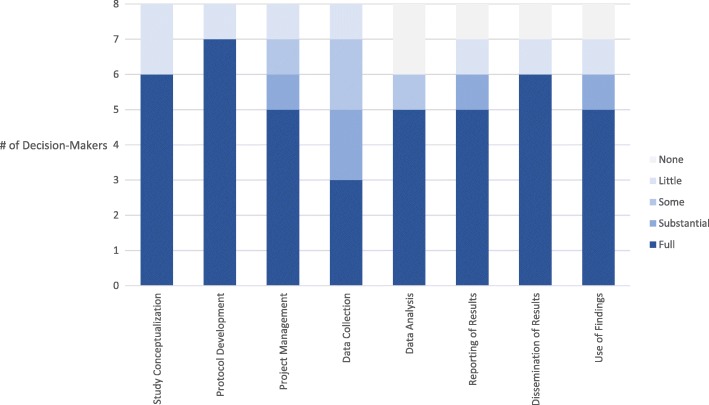
Engagement of decision-makers in the stages of embedded implementation research

The results of the questionnaire on decision-maker engagement in the embedded research approach are presented in Fig. 1. Responses showed strong engagement in protocol development (*n* = 6), moderate engagement in data collection (*n* = 3) and low engagement in data analysis (*n* = 5). According to the results of the questionnaire, almost two-thirds (*n* = 5) of decision-makers thought the study generated evidence that was useful for addressing the implementation barrier that programmes are facing. The majority (*n* = 5) of decision-makers felt that the options for action supported by the findings of the study were feasible to implement and could be used to improve health policies and programmes. Three-quarters (*n* = 6) were willing to consider the findings of the study when making decisions and/or were willing to advocate for the findings of the study to other decision-makers responsible for implementation, and the same proportion was willing to lead further research projects when facing implementation barriers.

Respondents reported that the embedded research approach substantially or fully built their research generation and use capacities, with the highest capacity improvements observed for ‘presenting research findings to policy and decision-maker audiences’ (*n* = 6), while improvements for ‘carrying out research on implementation’ and ‘elaborating options for action that are supported by the results’ were reported by four decision-makers. However, few respondents reported being ‘fully confident’ in research activities, with the greatest level of confidence reported for ‘carrying out research on implementation’ and ‘elaborating options for action that are supported by the results’. The least confidence was reported for ‘assessing failures within the health system’ and ‘bringing about changes in programmes and systems’.

### In-depth interview results

All stakeholders involved as co-principal investigators in the embedded research initiative were contacted to conduct in-depth interviews. Ten decision-makers (6 men and 4 women) were interviewed, including all respondents who completed the questionnaire. As outlined in Table 4, respondents were decision-makers working at national (*n* = 4), provincial or state (*n* = 5) and municipal (*n* = 1) level, and represented the following countries: Argentina, Bolivia, Brazil, Chile, Colombia, Mexico, Peru and Saint Lucia.

**Table 4:** In-depth interview respondent characteristics

Country	Affiliation	Level	Sex
Argentina	Direction of Child, Adolescence, Sexual and Reproductive Health, Ministry of Health of the Province of Santa Fe, Argentina.	Provincial	Male
Argentina	National Chagas Program	National	Female
Bolivia	El Alto Regional Health Service	Provincial	Female
Brazil	Specialized Assistance Services (SAS) of Ceará	Provincial	Female
Chile	Municipality of Huechuraba	Municipal	Female
Chile	Ministry of Health	National	Female
Colombia	The Colombian Health Technology Assessment Institute (IETS)	State	Male
Mexico	Centro Nacional de Excelencia Tecnologica en Salud (CENETEC-Salud) Servicios Estatales de Salud de Oaxaca	State	Male
Peru	Ministry of Health	National	Male
Saint Lucia	Ministry of Health	National	Female

Results of the in-depth interviews with decision-makers revealed 17 facilitators and 8 barriers to embedded implementation research projects and its application in policy and practice. The most common facilitating factors and barriers are assessed below.

### Facilitators

#### Actionability

Respondents from nine projects spoke about the actionability of the research. Six respondents spoke about using the research to inform a decision, proposal, plan or policy for implementation. One respondent said:

*“This project made it possible to find out whether what we are proposing will be good or not, or if there was a better way to do it.*” (Respondent from national health system level)

Four spoke about using the research to advocate for change or gain support:

“*The study allows us to produce evidence of where the difficulties are, the gaps at which this policy should aim, so that when we present the policy to the authorities* […]*, it is much better to have evidence to support and back up that policy, and we have been able to get this with the study results.*” (Respondent from national health system level)

#### Relevance

Eight respondents mentioned issues related to relevance as a driver of the research. One respondent said:

“*The* [embedded implementation research] *project* [generated] *the evidence that the programme needs* [to learn] *about how to change.*” (Respondent from national health system level)

According to another respondent:

“*Everyone agreed* [with the research] *because it was in line with the work that we were doing. The issue of implementation* […] *was bothering us a bit* […] *and this research came in exactly that* […] *moment* […] *so we agreed that this was the ideal combination between research and governance so as to guide the adjustments that had to be made in the implementation.*” (Respondent from provincial or municipal health system level)

#### Engagement

Eight respondents spoke about engagement of stakeholders; specifically, four described how the participation resulted in greater acceptability or openness to the research or change. Three respondents talked about engaging the necessary stakeholders to bring about change:

“*This research has made it possible for all these separate* [stakeholders] *to come to an agreement to improve.*” (Respondent from provincial or municipal health system level)

Maintaining the attention of the authorities was also mentioned by two respondents. In describing the engagement of high-level stakeholders, one respondent said:

“*I think* [engaging stakeholders] *is obviously how we have been working and that has made it possible to maintain the attention of the authorities*.” (Respondent from provincial or municipal health system level)

### Barriers

#### Time

Six respondents spoke about issues related to time. Three respondents spoke about the challenge of time in carrying out the implementation research. One respondent mentioned that the short timeframe for the projects caused the research to be restricted, while another discussed the time it took to receive ethical approval for the project causing delay. Notably, one respondent also shared the challenge of finding the time to carry out the research and balancing the extra activity with other programmatic responsibilities. Two respondents each spoke about the challenge of time related to bringing about changes or impact and/or reaching agreement among stakeholders. As one respondent said:

“*The time that we may need for chang*[ing] *their habits and the date they have to improve* […] *were limited.*” (Respondent from provincial or municipal health system level)

When describing the engagement of stakeholders in the research, another respondent stated:

“*It takes a bit of time because it is more demanding, for all of us to agree on one strategy.*” (Respondent from provincial or municipal health system level)

#### Political process

Four respondents identified issues related to working with the government. Of these, three respondents spoke about the challenge of turn-over and government changes affecting the carry out of the work or resulting in delays. As one respondent explains:

“*We don’t really know which are the interests of the new government, but this is really putting some new barriers that were not thought before because we were in the same line and the same objectives with the public health and different kinds of things…and now…*” (Respondent from national health system level)

Three also mentioned political influences in the government that affected the projects or use of the empirical results. In referring to how decisions are made in government, one respondent said:

“*There are a lot of factors that play into this kind of research and this kind of implementation of interventions, so it is difficult.*” (Respondent from national health system level)

## Discussion

Our assessment of the ten implementation studies conducted in the Latin America and Caribbean region provides key insights on the innovative embedded research approach tested in different health systems settings. Our study shows an overall satisfaction of decision-makers engaged in the implementation research process as well as a positive perception of embedding research in policy and systems decision-making. Across our study settings, satisfaction of policy-makers is associated with the actionability, relevance and context-sensitivity of research findings, perceived as a good fit-for-purpose to support health policy and programme implementation. The perceived usefulness of the embedded research approach in the Americas is consistent with previous evidence on the benefits of embedding research in real world policy- and decision-making in other settings [30, 31].

By promoting the active engagement of policy-makers from the onset, and by ensuring continuous policy engagement throughout the research, the embedded research model addresses two important barriers to the uptake of evidence — the engagement and ownership of end-users, and the applicability of relevant and contextualised research [2]. The embedded research approach puts forth an early engagement of policy-makers at the protocol development stage and stimulates a meaningful collaboration throughout the research cycle, thus contributing to the policy-relevance of research findings. As such, the embedded research approach in Latin American and Caribbean countries echoes previous experience showing that knowledge uptake to support health policies and systems is catalysed by on-going engagement in the research continuum as well as participation and trust among stakeholders [6, 32].

There is an increasing empirical base to support the co-production of research within the health sector, including the engagement of patients, communities, healthcare providers and health systems decision-makers [7, 8, 13, 21, 33–37]. The approach of embedding research into policy and practice also builds on the experience and methods put forth by the field of participatory action research. In addition, co-production of research has been spearheaded in other sectors, including environment, education and social welfare [38], speaking to its potential to advance the Sustainable Development Goals.

Engaging policy-makers in developing the research objectives and empirical questions ensures that research addresses key priorities and evidence needs for local health systems. The embedded component stimulates a demand-driven process, thus addressing recent calls for greater efforts to stimulate the generation of research questions by end-users [10, 11] and co-production of scientific evidence [9]. Furthermore, active engagement of decision-makers in the conduct of the study increases the relevance and practical application of the research outputs — both in terms of content and format — and the potential of research to bring about real-world improvements in health systems, which is a fundamental principle of implementation science [15]. In Argentina, for instance, a deliberative dialogue discussing the research findings provided recommendations to improve the implementation of the perinatal healthcare policy [20]. These collaborative processes are an opportunity to illuminate the relevance of research findings, explore the options for changes and improve the decision-maker’s perception on the usefulness of empirical evidence. Such hands-on and actionable evidence increases the likelihood that research is used to support real-world implementation and health systems strengthening.

### Addressing health systems failures

By documenting the systemic problems that are contributing to the suboptimal implementation of health interventions, the embedded studies also shed light on important systemic failures in health systems arrangements and performance. In Chile, for instance, the research underlines that suboptimal implementation of the national programme for clinical practice guidelines is associated to a lack of systemic and standardised processes for evidence-informed development and management of guidelines [21]. By identifying the underlying dysfunctions in healthcare services organisation, embedded research provides critical insights on correlations between implementation barriers and health systems failures in specific LMIC contexts. In addition, documenting the complex root causes and applying a health systems lens further addresses a critical knowledge gap to understand complex adaptive health systems in LMICs [39]. Yet, our study highlights the difficulty in enhancing capacity to apply systems thinking for health systems strengthening, and the need for further investments in capacity strengthening activities in this regard. Furthermore, in the context of embedded research, the time and resources required to explore systemic factors need to be balanced with the need to produce actionable results for implementation and policy improvements.

### Challenges for embedded research

Our study also shows that complex political and systems processes can at times act as an impediment to the uptake of embedded research findings. Although the scientific community is advocating for greater uptake of research in health policy and practice [10, 11], empirical findings remain only one component of complex decision-making processes, thus potentially limiting the impact of embedded research. Our study suggests that building consensus around implementation improvements informed by embedded research also requires intensive engagement and time from decision-makers, who are grappling with various competing interests. These findings corroborate the body of evidence highlighting the role of power dynamics as a critical factor in evidence-informed policy-making [40, 41].

In addition, our study highlights the need for realistic expectations of embedded research endeavours, as enhancing policy implementation and health systems strengthening require time and resources that often go beyond the lifecycle of the research. The scope of embedded research initiatives and the need to engage different stakeholders thus need to be carefully considered from the onset to strike a balance between timely improvements to the implementation of interventions and longer-term strengthening of health systems.

Furthermore, embedding research in policy and practice also raises important ethical and empirical questions about potential biases of decision-makers when assessing policy implementation or systems performance, which is a potential caveat of the embedded approach. To mitigate this process and uphold scientific validity of implementation research, our model showcases the engagement of researchers as co-principal investigators of the research project. Yet, there is a need to further document and study the dynamics of co-production schemes in health systems research, to inform good practices and to learn from the challenges in applying this approach in different contexts. The need for evidence thus pertains to greater evaluations of decision-maker engagement models in both primary research and evidence synthesis [42]. In turn, this evidence will inform further guidance on when and how to embed research in policy and practice, recognising that health systems investments also require external and independent evaluation of policy effectiveness and implementation in different circumstances [43].

### Way forward in embedding research in policy and systems

Our multi-country analysis shows that action-oriented research outputs and active engagement of knowledge-users contribute to a strong buy-in of stakeholders involved in health systems strengthening at national and sub-national levels. For instance, the implementation research findings identified by the team in Peru shed light on the fragmentation of healthcare for patients co-infected by tuberculosis and HIV [19]. In turn, the results informed the development of a comprehensive model for integrated healthcare services for this vulnerable population, which is now being scaled-up nationally with additional support from the Global Fund to Fight AIDS, Tuberculosis and Malaria. The decision-makers’ favourable perception of the embedded model, including potential return-on-investment for implementation research, is also aligned with previous experiences showing that modest costs associated with researching implementation barriers can generate a magnifier effect by extending the impact of health interventions [44].

As the knowledge base on embedded health systems research remains limited, our multi-country analysis contributes to the development of this priority research agenda for global health. As such, there is a need to better assess and document critical issues of management, leadership and championship, and how they might influence the use of knowledge to support policy and programme implementation. Further knowledge is also needed to understand the optimal approach, level and intensity of engagement of decision-makers, including policy-makers and programme managers, in the conduct of all research phases, considering the resources and time required for empirical work and the competing interests of daily implementation obligations. In addition, further research is needed on the pathways through which engagement and leadership of decision-makers lead to greater uptake of research in health systems. Previous research links stakeholder involvement to higher trust and confidence in research outcomes [45]. Value-based assessment of research priorities [3] and integration of experiential knowledge of end-users [46] have also been associated with evidence use, yet more research is needed to understand the mechanisms and determinants of effective engagement approaches to strengthen evidence-informed decision-making.

### Strengths and limitations

The strengths of this study include triangulation of the findings using different data sources and a reflexive member checking of results with the country participants, aiming to uphold data trustworthiness. A limitation of this multi-country analysis is the absence of critical appraisal of the implementation studies using validated tools. This embedded research experience in Latin America and the Caribbean reveals important challenges in the conduct and support of this type of empirical endeavour, not least pertaining to understanding the complexity and power dynamics of decision-making within health systems and government processes. Research addressing real-world contexts is inherently complex and requires intensive efforts to unpack and understand the political economy, structures and processes underlying health policies and programmes. Although some teams have addressed elements of complexity, for instance, Colombia’s stakeholder analysis for the implementation of clinical practice guidelines for sexually transmitted infections [18], most teams did not have sufficient time nor resources to thoroughly appraise the complex policy and health system settings. Further limitations of this study include the small sample size and a potential social desirability bias of answers provided during the in-depth interviews and questionnaires. In addition, the direct assessment of the findings by a WHO team might have influenced the interpretation of the data.

## Conclusion

Our study contributes to enhance the limited empirical and evaluative evidence base on co-production of health systems research, with a view of improving the implementation of health systems interventions. Findings from the embedded implementation research initiatives provide relevant and context-specific knowledge to improve the implementation of health policies and programmes, while shedding light on the underlying performance of health systems. Our multi-country analysis further contributes to strengthening the knowledge base for the embedded approach in the field of health policy and systems research. Our study provides support on the usefulness of embedding research in health policy and systems decision-making, adding to previous requests for greater stakeholder engagement and co-production of knowledge in health systems research [47, 48]. At the same time, it highlights the challenges and potential caveats of embedding research in complex policy and systems decision-making, and the need for capacity strengthening efforts, particularly in LMICs.

The embedded research experience in the Americas also shows the interest, political will and readiness to invest in active engagement and capacity strengthening models to conduct and use health policy and systems research. By changing the traditional modus operandi of health research, the embedded research approach puts forth an innovative way of developing science and engaging decision-makers in research. As such, embedding research in health policy and programme requires a change of mindsets in both researchers and health system decision-makers. This is critical, as embedding research into real world policy and practice bears the potential to improve implementation and scale-up of effective health interventions, thus contributing to the relevance of research to support universal health coverage schemes globally.

## Supplementary information

**Additional file 1.** In-depth interview questions.

**Additional file 2.** COREQ (COnsolidated criteria for REporting Qualitative research) Checklist.

## Data Availability

The datasets used and/or analysed during the current study are available from the corresponding author on reasonable request.

## Abbreviations

Alliance: Alliance for Health Policy and Systems Research
IECS: Instituto de Efectividad Clínica y Sanitaria
iPIER: Improving Programme Implementation through Embedded Research
LMIC: Low- and middle-income country

## References

[1] Bosch-Capblanch X (2012). Guidance for evidence-informed policies about health systems: rationale for and challenges of guidance development. PLoS Med.

[2] Oliver K (2014). A systematic review of barriers to and facilitators of the use of evidence by policymakers. BMC Health Serv Res.

[3] Deverka PA (2012). Stakeholder participation in comparative effectiveness research: defining a framework for effective engagement. J Comp Eff Res.

[4] Murphy K, Fafard P (2012). Taking power, politics, and policy problems seriously: the limits of knowledge translation for urban health research. J Urban Health.

[5] Greenhalgh T, Wieringa S (2011). Is it time to drop the ‘knowledge translation’ metaphor? A critical literature review. J R Soc Med.

[6] Langlois EV (2016). Enhancing evidence informed policymaking in complex health systems: lessons from multi-site collaborative approaches. Health Res Policy Syst.

[7] Boivin A (2018). Patient and public engagement in research and health system decision making: A systematic review of evaluation tools. Health Expect.

[8] Olivier J, Whyle E, Gilson L (2017). Embedded Health Policy and Systems Research: A Rapid Scoping Review.

[9] Greenhalgh T (2016). Achieving research impact through co-creation in community-based health services: literature review and case study. Milbank Q.

[10] World Health Organization. Research for Universal Health Coverage: World Health Report 2013. http://www.who.int/whr/2013/report/en/. Accessed 27 May 2019.

[11] World Health Organization. WHO Strategy on Health Policy and Systems Research: Changing Mindsets. 2012; http://www.who.int/alliance-hpsr/alliancehpsr_changingmindsets_strategyhpsr.pdf. Accessed 27 May 2019.

[12] Ryan M (2001). Eliciting public preferences for healthcare: a systematic review of techniques. Health Technol Assess.

[13] Langlois EV, Tran N, Ghaffar A, Reveiz L, Becerra-Posada F. Embedding research in health policy and systems in the Americas. Rev Panam Salud Publica. 2017;41:e68. 10.26633/RPSP.2017.68 PMC6612733 29069142

[14] Tran N (2017). Embedding research to improve program implementation in Latin America and the Caribbean. Rev Panam Salud Publica.

[15] Peters D, Tran N, Adams T. Implementation Research in Health: A Practical Guide. In: Alliance for Health Policy and Systems Research. Geneva: World Health Organization; 2013.

[16] Oliver K, Lorenc T, Innvaer S (2014). New directions in evidence-based policy research: a critical analysis of the literature. Health Res Policy Syst.

[17] Bawah AA (2019). The child survival impact of the Ghana Essential Health Interventions Program: A health systems strengthening plausibility trial in Northern Ghana. PLoS One.

[18] Moreno JHR (2017). Evaluation of tools for the implementation of clinical practice guidelines on sexually transmitted infections. Rev Panam Salud Publica.

[19] Garcia-Fernandez L, Benites C, Huaman B (2017). Access barriers to comprehensive care for people affected by tuberculosis and human immunodeficiency virus coinfection in Peru, 2010–2015. Rev Panam Salud Publica.

[20] Simioni AT (2017). Regionalization of perinatal health care in the province of Santa Fe, Argentina. Rev Panam Salud Publica.

[21] Herrera P (2017). Clinical practice guidelines: qualitative study of their implementation in the Chilean health system. Rev Panam Salud Publica.

[22] Klein K (2017). Strategy to improve access to etiological treatment of Chagas disease at the first level of care in Argentina. Rev Panam Salud Publica.

[23] Tinajeros F (2017). Health-worker barriers to syphilis screening in pregnant women in Bolivia's Los Andes network. Rev Panam Salud Publica.

[24] Velazquez M (2017). Evaluation of the teleconsultation process from the perspective of the provider (Oaxaca Telehealth Program, Mexico). Rev Panam Salud Publica.

[25] Ramirez GR (2017). Adolescents’ access to contraception: perceptions of health workers in Huechuraba, Chile. Rev Panam Salud Publica.

[26] de Paula SHB (2017). Evaluation of implementation of the protocol for managing tuberculosis/ human immunodeficiency virus coinfection in specialized care services in ceara state. Rev Panam Salud Publica.

[27] Alexander S (2017). Knowledge of and attitudes toward heel prick screening for sickle cell disease in Saint Lucia. Rev Panam Salud Publica.

[28] Vaismoradi M, Turunen H, Bondas T (2013). Content analysis and thematic analysis: Implications for conducting a qualitative descriptive study. Nurs Health Sci.

[29] Braun V, Clarke V (2006). Using thematic analysis in psychology. Qual Res Psychol.

[30] Vindrola-Padros C (2016). The role of embedded research in quality improvement: a narrative review. BMJ Qual Saf.

[31] Tugwell P, Knottnerus JA (2017). Benefits of embedding researchers in a health service setting. J Clin Epidemiol.

[32] Keown K, Van Eerd D, Irvin E (2008). Stakeholder engagement opportunities in systematic reviews: knowledge transfer for policy and practice. J Contin Educ Heal Prof.

[33] Concannon TW (2014). A systematic review of stakeholder engagement in comparative effectiveness and patient-centered outcomes research. J Gen Intern Med.

[34] Domecq JP (2014). Patient engagement in research: a systematic review. BMC Health Serv Res.

[35] Shippee ND (2015). Patient and service user engagement in research: a systematic review and synthesized framework. Health Expect.

[36] Ghaffar A (2017). Strengthening health systems through embedded research. Bull World Health Organ.

[37] Peters DH, Bhuiya A, Ghaffar A (2017). Engaging stakeholders in implementation research: lessons from the Future Health Systems Research Programme experience. Health Res Policy Syst.

[38] Olivier JSV, Molosiwa D, Gilson L, Baker K, Dd S, Adam T (2017). Embedded systems approaches to health policy and systems research. In: Applied Systems Thinking for Health Systems Research: A Methodological Handbook.

[39] de Savigny D, Adam T (2009). Systems Thinking for Health Systems Strengthening.

[40] Erasmus E, Gilson L (2008). How to start thinking about investigating power in the organizational settings of policy implementation. Health Policy Plan.

[41] Sriram V (2018). 10 best resources on power in health policy and systems in low- and middle-income countries. Health Policy Plan.

[42] Tricco AC (2018). Engaging policy-makers, health system managers, and policy analysts in the knowledge synthesis process: a scoping review. Implement Sci.

[43] Peters DH (2018). Health policy and systems research: the future of the field. Health Res Policy Syst.

[44] Hales S (2016). Reporting guidelines for implementation and operational research. Bull World Health Organ.

[45] Burton H (2009). Developing stakeholder involvement for introducing public health genomics into public policy. Public Health Genomics.

[46] Saunders C (2007). Operationalising a model framework for consumer and community participation in health and medical research. Aust New Zealand Health Policy.

[47] Boote J, Telford R, Cooper C (2002). Consumer involvement in health research: a review and research agenda. Health Policy.

[48] Elwyn G (2010). Identifying and prioritizing uncertainties: patient and clinician engagement in the identification of research questions. J Eval Clin Pract.

