# Supplementary material for: Embedding implementation research to enhance health policy and systems: a multi-country analysis from ten settings in Latin America and the Caribbean
Source: Health Res Policy Syst. 2019 Oct 15;17:85. doi: 10.1186/s12961-019-0484-4 (PMC6794825; doi:10.1186/s12961-019-0484-4)
Supplement: Supplementary file 1 — Additional file 1. In-depth interview questions. [file 12961_2019_484_MOESM1_ESM.docx]

**Additional file 1.**

**In-Depth Interview Questions**

| **#** | **Question** |
| --- | --- |
| 1 | How did the idea for the study come about? |
| 2 | What were the major tasks of the decision-maker in the project? What were the major tasks of the researcher in the project? |
| 3 | What capacities do you feel were strengthened during the period of the study? |
| 4 | From your experience, what are the advantages and disadvantages of the embedded approach to research, i.e. research led by a decision-maker? |
| 5 | How was the health system considered in the design of the study? How may the findings generated by the study be used to strengthen the health system? |
| 6 | How have the results of the study been received by decision-makers? What factors facilitated or hindered this reception? |
| 7 | What changes have you seen as a result of the study?  In programme implementation? Within the health systems? Within the Ministry of Health or implementing organization? |
| 8 | How has your attitude towards research changed? Do you have any plans to pursue or carry out implementation research in the future? |
